# Supplementary material for: Effects of an equol-containing supplement on advanced glycation end products, visceral fat and climacteric symptoms in postmenopausal women: A randomized controlled trial
Source: PLoS One. 2021 Sep 10;16(9):e0257332. doi: 10.1371/journal.pone.0257332 (PMC8432832; doi:10.1371/journal.pone.0257332)
Supplement: S1 File — (PDF) [file pone.0257332.s001.pdf]

Relationship between equol producer status and intestinal microflora and effects of supplementation with equol and lactobionic acid on metabolic and bone parameters in healthy postmenopausal women

Research contact person
Yoko Ishigaki
Shinkokai Medical Corporation, Sendai General Health Screening Clinic
Sendai Trust Tower 4F, 1-9-1 Ichiban-cho, Aoba-ku, Sendai-shi, Miyagi prefecture
Tel：022-722-3770
FAX：022-221-0020

Remi Yoshikata
Midtown Clinic Medical corporation, Hamamatsucho Hamasite Clinic
Shiodome Building 2F, 1-52-20 Kaigan, Minato-ku, Tokyo
Tel：03-5472-1100
FAX：03-5472-3355
Version: June 26, 2017

Outline of Research

1. Objective

To investigate the relationship between dietary status, type of intestinal bacteria, and equol-producing ability.

To examine the effects of ingestion of "equol + lactobionic acid" on various parameters of lifestyle-related disease risk such as arteriosclerosis and visceral fat area including the relationship with the above background factors.

2. Research population

Postmenopausal women who undergo medical examinations at the Sendai General Health Screening Clinic.

3. Methods

Subjects: Healthy postmenopausal women aged 50 years or older who undergo a medical examination at the above facility and who have consented to this study.

Period: August 2017-February 2018

Supplement: "Equol-containing fermented soybean" and "Lactobionic acid-containing fermented lactose" foods(Contains S-form equol 10 mg / day and lactobionic acid 150 mg / day)

Survey contents: Equal production ability test by urinalysis, intestinal bacteria test by stool test, eating habit assessment survey by self-administered questionnaire, menopausal symptom questionnaire, general biochemical test by blood sampling, visceral fat area measurement by visceral fat CT test (fat scan) , AGE value measurement by AGE reader, pulse wave velocity measuremnt

Timing of investigations: At the start of the survey, and every month, at the end of the survey (12 weeks later)

4. Research period and estimated sample size

Research period: August, 2017 to February 2018

Estimated sample size: 60 women

5. Research location

Shinkokai Medical Corporation, Sendai General Health Screening Clinic

Contents

1. Research Objective and Background 5
2. Research Population 5
3. Method 6
4. Measures 6
5. Outcome evaluation 6
6. Schedule 7
7. Analysis 7
8. Expected adverse events 7
9. Handling of adverse events 7
10. Medical expenses and compensation 9
11. Ethical issues 9
12. Research Location 11
13. Principal researchers 11
14. Perosn in-charge of personal information management 11
15. Persons in-charge of explanation on clinical trial....11
16. Clinical Research Office 11
17. Test kits 11
18. Providing supplement 11
19. Announcement of research results 11
20. Budget 11
21. References 125

June 26, 2017

Outline of clinical trial on equol and lactobionic acid supplement

1. Research Objective and Background

Equol is an active metabolite in which daidzein, a type of soy isoflavone, is metabolized by intestinal bacteria. Its structure is similar to that of the female hormone estrogen, and it has an estrogen-like action, which alleviates menopausal symptoms. , Suppression of bone loss, improvement of lipid metabolism and arteriosclerosis, etc. have been reported, and are expected to be effective for a wide range of women's health care.

On the other hand, the number of people who can produce equol in the body is as small as about 50% in Japan, and it is said that the eating habits, living environment, and intestinal bacteria, which are equol-producing bacteria, are involved in the equol-producing ability.

In this clinical study, it is considered meaningful to investigate and clarify the relationship between dietary habits and the types of intestinal bacteria regarding equol-producing ability.

In addition, we investigated the effects of ingestion of "equol + lactobionic acid" on lifestyle-related disease parameters such as vascular system, visceral fat area, and AGEs (advanced glycation end products) in the above subjects, and investigated the effects of equol production ability and intestinal bacteria It is important to clarify the relationship with the background factors of equol in order to further build evidence on the action of equol.

2. Research Population

1) Target person

Those who have recruited subjects from postmenopausal* healthy women aged 50 years or older who wish to undergo a medical examination at the Sendai General Medical Examination Clinic and have obtained their consent to participate in this study.

* Menopause is spontaneous menopause, excluding menopause due to invasion such as surgery.

2) Selection, exclusion, discontinuation criteria

①Selection criteria

Those who meet all of the following selection criteria and have the ability to consent are eligible.

・Japanese healthy postmenopausal women over 50 years old

・Persons undergoing medical examination at Sendai General Clinic

・Persons who can undergo interviews or examinations at the start of the examination and every month at the Sendai General Clinic (8 minutes on foot from JR Sendai Station)

・Person who can take the recommended quantity of supplement and record daily life diary during the implementation period (12 weeks)

②Exclusion criteria

Persons with a history of allergies to soy foods, dairy products, and brewer's yeast.

・Persons who have started taking medicines or health foods that may have an effect during the test period.

・Those who are receiving HRT or who are taking drugs that may act on hormones.
・Others that the investigating doctor deems inappropriate.
③Cancellation criteria
Clinical research will be discontinued if any of the following applies.
・ During the test period, the subject develops serious symptoms and the safety of the supplement becomes questionable.
・ When it becomes clear that the subject was affected by continuously consuming the supplement.

3) Registration
・The principal investigator will register the candidate as a subject after confirming that she meets the eligibility criteria and does not meet any of the exclusion criteria.

3. Method

1) Survey period: 12 weeks (August 2017-February 2018)
2) Test method: Epidemiological survey, clinical trial (randomized open trial)
3) Number of cases: 60 cases (30 women who take equol + lactobionic acid supplement, and 30 women who do not take the supplement)
4) Test food: "Equol + lactobionic acid" 3 tablets (10 mg) daily (marketed in December 2013)

4. Measures

Before ingesting the supplement: Equol check (in urine), gut microbiota test (stool test), dietary habit assessment (BDHQ).
Before and after ingestion of supplement: height / weight / body composition, blood test (TG, HDL-C, LDL-C, T-CL, uric acid, HbA1C), baPWV, visceral fat CT test (fat scan), AGE measurement by AGE reader, and climacteric system assessments.

5. Outcome evaluation

1) Primary Outcomes: blood test (TG, HDL-C, LDL-C, T-CL, uric acid, HbA1C), baPWV, visceral fat area
2) Key Secondary Outcomes: AGE, relationship between equol-producing ability, type of intestinal bacteria, and dietary habit assessment (BDHQ) from the test results, safety of supplement, climacteric symptoms

6. Schedule

	One week before	Week 0	Week 4	Week 8	Week 12	Result report
Explanation and consent	◎					
Urine equol test		○				◎
Intestinal flora test		○				◎
Dietary questionnaire		○				◎
Blood test		○			○	◎
baPWV		○			○	◎
Body weight measurement		○			○	
Visceral fat scan		○			○	◎
AGE reader		○			○	◎
Supplement		←	→	→	→	
Climacteric symptom assessment		○	○	○	○	
Safety monitoring		○	○	○	○	

7. Analysis

For the primary measures, perform chi-squared test and t test for comparison between groups and before and after the study period. For secondary outcomes, anonymized data are entered into the database and the correlation is analyzed. Drop out cases and missing values are not included in the analysis. Other questionnaire surveys will be conducted according to the evaluation criteria of each questionnaire. The visceral fat CT examination and baPWV measurement at the start of the test and at 12 weeks will be performed at the same site in order to eliminate measurement errors.

8. Expected adverse events

In rare cases, allergic symptoms to supplement may appear.

9. Handling of adverse events

1) Symptoms or disease

Any unfavorable or unintended signs, symptoms or disease that develops during the study is treated as an adverse event. However, worsening of biomedical parameters in the study will not be treated as an adverse event.

2) Objective findings

If abnormalities or deterioration (normal → abnormal / worse, abnormal / worse → more abnormal / worser) are detected after the study period compared to before the study, it is treated as an adverse event. Regardless of the items specified or unspecified in this protocol, those considered to be adverse events need to be recorded in the case report, describing the onset, time of maximum severity, outcome, and relevance.

3) Recording and investigation of adverse events

If an adverse event occurs, its symptoms or disease, content of objective findings, date of onset, degree,

severity, presence or absence of treatment and its content, outcome and date of diagnosis, relevance to this clinical study and the underlying reason are to be recorded in the adverse event section of the case report. When the name of the disease is described, the symptom associated with the disease is not recorded as an adverse event.

If an adverse event is observed in the symptoms, diseases, or objective findings observed during the treatment period, it will be followed up until the condition become normalized or improved up to a level that could not be regarded as an adverse event, regardless of whether it has a causal relationship with this clinical study. However, this does not apply if the investigator determines that the patient has recovered. In that case, the grounds for determining recovery shall be stated in the case report form. If irreversible adverse events are observed due to organic disorders (cerebral infarction, myocardial infarction, etc.), follow-up will be conducted until the symptoms stabilize or improve.

4) Classification of adverse events

The degree of adverse events is classified according to the following criteria.

① Mild: To the extent that the subject's daily life is not impaired

② Moderate: It interferes with the subject's daily life, but if he / she puts up with it, he / she can carry out the activity.

③Altitude: Degree that greatly interferes with the subject's daily activities

Adverse event outcomes are classified according to the following criteria:

① Recovery: Namalization or recovery to a level that cannot be regarded as an adverse event

②Continuation: Those that have not recovered at that time

③Unknown (death): The outcome was unknown due to the death of the patient

5) Judgment of the relationship between adverse events and this clinical study

The relevance to this clinical study will be determined according to the following relevance criteria, taking into consideration the condition of the subject, the time relationship with treatment, and the possibility of other factors.

①Obviously related

② Probably related

③ May be related

④ Not related

Regarding adverse events, items ①to ③) are those whose relevance to this clinical study cannot be ruled out, and item ④is regarded as an adverse event whose relevance to this clinical study is denied.

6) Serious adverse events

If a serious adverse event occurs during the study period, whether or not it has a causal relationship with this clinical study, the investigator will immediately give an appropriate treatment to the subject.

7) Providing new information

When new information on the safety of this clinical study is obtained, the explanatory document and consent document will be revised promptly as necessary.

10. Medical expenses and compensation

1) Medical expenses

・ The cost of the first regular medical examination will be borne by the patient, and if it is clear that it is related to this clinicaltrial, the patient will not be liable for the cost. If it is unclear, it will be decided through consultation.

・ If medical expenses are incurred, the research implementation facility, Sendai General Health Examination Clinic, and Advanced Medical Care Co., Ltd., the sponsor company, will deal with them upon discussion.

2) Compensation

If the subject is liable for compensation as a result of health damage due to this clinical study, the investigator and the sponsor company will discuss and decide how to handle it.

11. Ethical issues

1) Compliance with ethical guidelines for clinical research

This clinical study will be conducted in compliance with the ethical principles based on the "Ethical Guidelines for Clinical Research" (Ministry of Health, Labor and Welfare) and this protocol.

2) Ethics Committee

The ethical issues and validity of this proposal will be discussed in the Institutional Review Board of the Medical Corporation of Shinkokai. The trial must be conducted according to the protocol approved by the committee.

①When a serious adverse event occurs

②When significant changes are made to the protocol

③When there is a significant change in the consent and other explanatory documents

④When other documents related to the protocol are revised

3) How to obtain the consent of the subject

Prior to the start of this clinical study, the principal investigator will explain the following matters to the subject based on the explanatory document, and then obtain the written consent of the subject based on his / her free will.

The following items should be described in the explanatory document.

① Participation in the trial

②Purpose of clinical research

③Clinical research method (including experimental aspects, subject selection criteria, etc.)

④Scheduled period of participation of subjects in clinical research

⑤ Number of subjects planning to participate in clinical research

⑥Expected clinical benefits and risks or inconveniences (if there is no expected clinical benefits of the subject, inform them)

⑦Presence or absence of other treatment methods when using patients as subjects, and the significant benefits and risks predicted regarding the treatment methods.

⑧Treatments that subjects can receive in the event of clinical research-related health hazards

⑨Participation in clinical research is voluntary, and the subject may refuse or withdraw

participation in clinical research at any time. In addition, refusal or withdrawal will not result in any disadvantage to the subject or loss of benefits that would be incurred if the subject did not participate in the clinical study.

⑩Promptly inform the subject of the continuation of participation in the clinical study when information that may affect the intention of the subject or his / her substitute is obtained.

⑪Conditions or reasons for discontinuing participation in clinical research

⑫Monitors, auditors, clinical research review committees, and domestic and foreign regulatory authorities can view the source materials such as medical records. At that time, the personal information of the subject should be protected. In addition, the documents can be allowed to view if the subject has already stamped or signed the consent document.

⑬The personal information of the subject should be protected even when the results of clinical research are published.

⑭the detailed information if the subject needs to bear the cost

⑮The name, job title and contact information of the principal doctor in-charge of this trial or clinical research collaborator (coordinator)

⑯Medical institution consultation desk to contact if the subject wants more information regarding clinical research and rights of the subject

⑰Responsibilities of the subject

4) Providing information to subjects

When the investigator obtains information that may affect the subject's will regarding the continuation of participation in the clinical study, the investigator promptly informs the subject or his / her substitute to participate in the clinical study, and check if the subject wants to continue. In addition, this process will be recorded in the medical record.

5) Protection of personal information

Protect the personal information of subjects when conducting clinical trials.

In protecting personal information, all data related to this protocol should be anonymized (coded) and handled. Information that can be identifiable personally will be stored and managed in a place where it can be locked under the strict control of the person in-charge of personal information management at the Sendai General Medical Examination Clinic. When it is no longer needed, dispose of it in an irreproducible form such as incineration or dissolution. In the unlikely event that personal information is leaked, the following measures will be taken promptly.

-Fact-finding, investigation of cause

-Identify the range of influence

-Examination and implementation of recurrence prevention measures

-Contacting potential affected individuals

-Report to the Minister of Health, Labor and Welfare

-Publication of facts, recurrence prevention measures, etc.

12. Research Location

Shinkokai Medical Corporation, Sendai General Health Screening Clinic

13. Principal researchers

Yoko Ishigaki (Shinkokai Medical Corporation, Sendai General Health Screening Clinic)
Remi Yoshikata (Midtown Clinic Medical Corporation, Hamamatsucho Hamasite Clinic)

14. Perosn in-charge of personal information management

Yuki Kawasaki (Manager, Sendai General Affairs, Shinkokai Medical Corporation)

15. Persons in-charge of explanation on clinical trial

Emiko Kikuchi (Registered Dietician, Sendai Planning Section, Sendai General Health Screening Clinic)
Ritsuko Sato (Public health nurse, Occupational Health Department, Sendai General Health Screening Clinic)

16. Clinical Research Office

Megumi Kato (Sendai Planning Section, Sendai General Health Screening Clinic)

17. Test kits

Equol check: Healthcare Systems Co. Ltd.
Fecal microflora test：Mykinso (Cykinso Co. Inc.)
AGEs：AGE READER MU (Selista Inc.)

18. Providing supplement

Advanced Medical Care Co. Ltd. provides equol plus lactobionic acid supplement.

19. Announcement of research results

When presenting the research results obtained in this study, the personal information of the subjects will be protected.

20. Budget

21. References

・Yoshikata R, et al, Relationship between equal producer status and metabolic parameters in 743 Japanese women: equal producer status is associated with antiatherosclerotic conditions in women around menopause and early postmenopause. Menopause 2017; 24, No2,216-224.

・Ishikawa N, et al, New equal supplement for relieving menopausal symptoms; randomized, placebo-controlled trial of Japanese women. Menopause 2009; 16: ４1-148.

・Usui T, et al, Effects of natural E-equal supplements on overweight or obesity and metabolic syndrome in the Japanese, based on sex and equal status. Clin Endcrinol 2013; 78:365-372.

・Yoshizumi T, et al. Abdominal fat: standardized technique for measurement at CT. Radiology 1999; 211; 283-286.
