# Supplementary material for: Effects of an equol-containing supplement on advanced glycation end products, visceral fat and climacteric symptoms in postmenopausal women: A randomized controlled trial
Source: PLoS One. 2021 Sep 10;16(9):e0257332. doi: 10.1371/journal.pone.0257332 (PMC8432832; doi:10.1371/journal.pone.0257332)
Supplement: S2 File — (PDF) [file pone.0257332.s002.pdf]

「閉経後健常女性におけるエクオール産生能と腸内細菌の関係および エクオール+ラクトビオン酸摂取における各種生活習慣病パラメータへの 影響に関する検討」

研究責任者
石垣洋子
医療法人社団進興会せんだい総合健診クリニック院長
住所：仙台市青葉区一番町 1-9-1　仙台トラストタワー4F
電話番号：022-722-3770
FAX 番号：022-221-0020

吉形玲美
医療法人ミッドタウンクリニック 浜松町ハマサイトクリニック
住所：東京都港区海岸 1-52-20　汐留ビルディング 2F
電話番号：03-5472-1100
FAX 番号：03-5472-3355

制作 2017 年 6 月 26 日　第二版

研究の概要

1. 目的

食生活の状況と腸内細菌の種類及びエクオール産生能との関係を調査する。
また、「エクオール+ラクトビオン酸」の摂取による動脈硬化、内臓脂肪面積など生活習慣病リスクの各種パラメータへの影響を上記背景因子との関連を含め比較検討する。

2. 研究対象者

せんだい総合健診クリニックで健診を受診する閉経後の女性。

3. 研究方法

対象者：上記施設にて健診を受診する 50 歳以上の閉経後健常女性で、本研究に同意を得た者

期間：2017 年 8 月～2018 年 2 月

被験食品：「エクオール含有大豆胚芽抽出発酵物」及び「ラクトビオン酸含有乳糖発酵物」食品
（S 体エクオールとして 10mg/日、ラクトビオン酸として 150mg/日を含有）

調査内容：尿検査によるエクオール産生能検査、便検査による腸内細菌検査、自記式アンケートによる食習慣アセスメント調査、更年期症状アンケート、採血による一般生化学検査、内臓脂肪 CT 検査（ファットスキャン）による内臓脂肪面積測定、簡易 AGE 検査による AGE 値測定、脈波伝播速度

調査時期：調査開始時、及び 1 ヶ月毎、調査終了時（12 週後）

4. 研究期間及び予定証例数

調査期間：2017 年 8 月～2018 年 2 月（12 週間）
予定症例：60 例

5. 研究実施施設

医療法人社団進興会 せんだい総合健診クリニック

目　次

1.　本研究の目的・背景　・・・・・・・・・・・・・・・・・・・・・・・・・・・・・Ｐ5
2.　研究対象者　・・・・・・・・・・・・・・・・・・・・・・・・・・・・・・・Ｐ5
3.　試験方法　・・・・・・・・・・・・・・・・・・・・・・・・・・・・・・・・Ｐ6
4.　検査内容　・・・・・・・・・・・・・・・・・・・・・・・・・・・・・・・・Ｐ6
5.　評価項目　・・・・・・・・・・・・・・・・・・・・・・・・・・・・・・・・Ｐ6
6.　試験スケジュール　・・・・・・・・・・・・・・・・・・・・・・・・・・・・Ｐ6
7.　評価方法　・・・・・・・・・・・・・・・・・・・・・・・・・・・・・・・・Ｐ7
8.　予想される有害事象　・・・・・・・・・・・・・・・・・・・・・・・・・・・Ｐ7
9.　有害事象の取扱い　・・・・・・・・・・・・・・・・・・・・・・・・・・・・Ｐ7
10. 医療費の負担と補償　・・・・・・・・・・・・・・・・・・・・・・・・・・・Ｐ8
11. 倫理的事項　・・・・・・・・・・・・・・・・・・・・・・・・・・・・・・・Ｐ8
12. 試験実施施設　・・・・・・・・・・・・・・・・・・・・・・・・・・・・・・Ｐ10
13. 試験実施責任医師　・・・・・・・・・・・・・・・・・・・・・・・・・・・・Ｐ10
14. 個人情報管理責任者　・・・・・・・・・・・・・・・・・・・・・・・・・・・Ｐ10
15. 検査キット　・・・・・・・・・・・・・・・・・・・・・・・・・・・・・・・Ｐ10
16. 披験食品の提供　・・・・・・・・・・・・・・・・・・・・・・・・・・・・・Ｐ10
17. 研究成果の発表　・・・・・・・・・・・・・・・・・・・・・・・・・・・・・Ｐ10
18. 費用概算　・・・・・・・・・・・・・・・・・・・・・・・・・・・・・・・・Ｐ11
19. 参考文献　・・・・・・・・・・・・・・・・・・・・・・・・・・・・・・・・Ｐ11

2017年6月26日

エクオール+ラクトビオン酸（サプリメント）の臨床研究概要

1. 本研究の目的・背景

エクオールは大豆イソフラボンの一種であるダイゼインが腸内細菌によって代謝された活性代謝産物で、その構造は女性ホルモンであるエストロゲンと類似しており、エストロゲン様作用を有すことから、更年期諸症状の緩和、骨量減少の抑制、脂質代謝及び動脈硬化の改善などが報告され、幅広い女性のヘルスケアへの効果が期待されている。

一方、エクオールを体内で産生できる人は、本邦において50%程度と少なく、エクオール産生能は、食生活、生活環境、さらにはエクオール産生菌である腸内細菌が関与していると言われている。

今回の臨床研究により、エクオール産生能について食生活の状況と腸内細菌の種類との関連を調査し明らかにすることは意義があると考えられる。

また、上記の被験者を対象に「エクオール+ラクトビオン酸」の摂取による、血管系や内臓脂肪面積、AGEｓ（糖化蛋白）など生活習慣病パラメータへの影響を調査し、エクオール産生能や腸内細菌との背景因子との関連を明らかにすることは、エクオールの作用におけるエビデンスをさらに構築する意味で重要と考える。

2. 研究対象者

1）対象者

せんだい総合健診クリニックで健診を希望する50歳以上の閉経後※の健常女性から被験者を募集し、本試験の参加に同意を得た者。

※　閉経は自然閉経とし、手術等の侵襲による閉経は除く。

2）選択、除外、中止基準

①　選択基準

下記の選択基準を全て満たし、かつ同意能力を有する者を対象とする。

・日本人の50歳以上の閉経後の健常女性

・せんだい総合クリニックで健診を受診する者

・試験開始時及び1ヶ月毎にせんだい総合クリニック（JR仙台駅から徒歩8分）にて問診または検査をうけることができる者

・実施期間（12週間）において所定量の被験食の摂取、及び日々の生活日誌の記録ができる者

②　除外基準

・大豆食品、乳製品、ビール酵母によるアレルギーの既往歴がある者。

・試験期間中に効果をもたらしうる医薬品の服用または健康食品の摂取を開始した者。

・HRTを受けている者、ホルモンに作用する可能性がある薬剤を服用している者。

・その他、試験実施医師が適切でないと認めたもの。

③　中止基準

下記に該当する場合は、臨床研究を中止する。

・試験期間中、被験者に重篤な症状が発症し、被験食品の安全性に問題が生じた場合。

・被験食の継続飲用により、被験者が何らかの不利益を被ることが明らかになった場合。

3）登録

研究責任者は、候補となる被験者が適格基準を満たし、除外基準のいずれに該当しないことを確認した上で、被験者として登録する。

3. 試験方法

1）調査期間：12 週間（2017 年 8 月～2018 年 2 月）

2）試験方式：疫学調査、臨床試験（ランダム化オープン試験）

3）症例数：60 例（エクオール+ラクトビオン酸摂取者 30 例、非摂取者 30 例）

4）被験食品：「エクオール+ラクトビオン酸」1 日 3 粒（10mg）（2013 年 12 月発売）

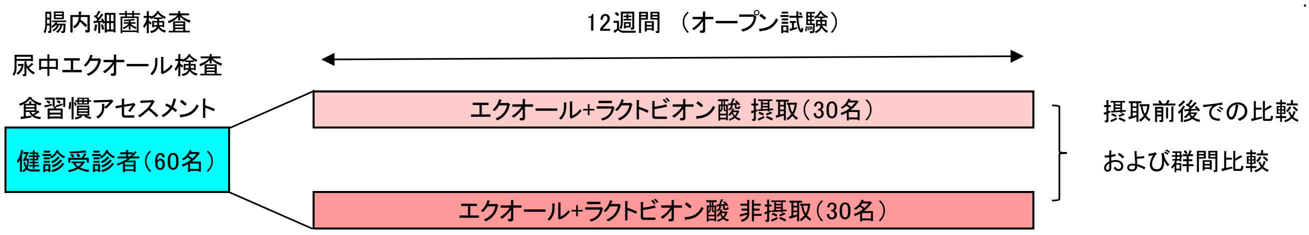

4. 検査・調査内容

抜験食品摂取前：エクオールチェック（尿中）、腸内細菌検査（便検査）、食習慣アセスメント（BDHQ）。

抜験食品摂取前後：身長・体重・体組成、血液検査（TG、HDL-C、LDL-C、T-CL、尿酸、HbA1C）、baPWV、内臓脂肪 CT 検査（ファットスキャン）、AGE 値（簡易検査）測定。更年期症状アンケート。

5. 評価項目

1）主要評価項目：血液検査（TG、HDL-C、LDL-C、T-CL、尿酸、HbA1C）、baPWV、内臓脂肪面積

2）副次的評価項目：AGE、エクオール産生能の有無と腸内細菌の種類、食習慣アセスメント（BDHQ）との関係を検査結果から評価する。抜験食品の安全性。更年期症状。

6. 試験スケジュール

	-1週	0週	4週	8週	12週	結果説明
事前説明・同意	◎					
エクオール検査（尿）		○				◎
腸内細菌検査（便）		○				◎
食習慣アンケート		○				◎
血液検査		○			○	◎
baPWV		○			○	◎
体重測定		○			○	
内臓脂肪CT検査		○			○	◎
AGE測定		○			○	◎
サプリメント摂取		◀	――	――	▶	
更年期症状アンケート		○	○	○	○	
安全性		○	○	○	○	

7. 評価方法

主要評価項目については群間比較、前後比較にてx2検定、T検定を行う。副次的評価項目については、匿名コード化した検査値をデータベースに入力し相関関係を分析する。中止・脱落症例、欠測値については、解析対象に含まない。他アンケート調査については、各アンケートの評価基準に従って実施する。試験開始時と12週時の内臓脂肪CT検査、baPWVの測定については、測定の誤差を無くすために同一部位で行うこととする。

8. 予想される有害事象

被健食品によるアレルギー症状がまれに現れる可能性がある。

9. 有害事象の取扱い

1）症状または疾患

試験中に発現した、あらゆる好ましくないあるいは意図しない徴候、症状または疾患は、有害事象として取り扱う。なお、有効性評価指標の程度が悪化した場合は、有害事象として扱わない。

2）他覚所見

臨床研究開始時検査値または評価と比較し、最終検査・評価日までに、異常化または悪化(正常→異常/悪化、異常/悪化→さらに異常/悪化)を示した場合は、有害事象として取扱う。

本研究実施計画書に規定された項目、規定されていない項目を問わず、有害事象とされたものについては、発現時、最大悪化時、転帰判定時及び関連性の判定に必要と考えられたデータについて症例報告書に記載する。

3）有害事象の記録と調査

有害事象が発現した場合は、その症状または疾患、他覚所見の内容、発現日、程度、重篤度、処置の有無およびその内容、転帰およびその判定日、本臨床研究との関連性およびその理由を症例報告書の有害事象欄に記載する。なお、疾患名を記載する場合、その疾患に付随する症状は、有害事象として記載しない。

治療期中に観察された症状または疾患、他覚所見において、有害事象が認められた場合は、本臨

床研究との因果関係の有無に係わらず、原則として正常化または有害事象として促えないレベルに回復するまで追跡調査を行う。ただし、研究責任医師が回復と判断した場合はその限りではない。その場合は回復と判断した根拠を症例報告書に記載するものとする。器質的な障害（脳梗塞・心筋梗塞など）で不可逆的な有害事象が認められた場合は、症状が安定または固定するまで追跡調査を行うこととする。

4）有害事象の分類

有害事象の程度は、以下の基準で分類する。

① 軽度：被験者の日常生活を損なわない程度

② 中等度：被験者の日常生活に支障があるが、かなり我慢すれば活動が行える程度

③ 高度：被験者の日常生活の遂行を大きく妨げる程度

有害事象の転帰は、以下の基準で分類する。

① 回復：正常化または有害事象として促えないレベルまでに回復したもの

② 継続：その時点で回復に至っていないもの

③ 不明（死亡）：患者死亡のため転帰が不明だったもの

5）有害事象と本臨床研究との関連性の判定

本臨床研究との関連性は、被験者の状態、治療との時間関係、その他の要因による可能性等を勘案し、以下の関連性の判定基準に従い判定する。

①明らかに関連あり

②おそらく関連あり

③関連があるかもしれない

④関連なし

有害事象については、本臨床研究との関連性が①～③と判定されたものを本臨床研究との関連性が否定できない有害事象、本臨床研究との関連性が④と判定されたものを本臨床研究との関連性が否定できる有害事象とする。

6）重篤な有害事象

試験期中に、本臨床研究との因果関係の有無にかかわらず重篤な有害事象が発現した場合、研究責任医師は、被験者に対して直ちに適切な処置を行う。

7）新たな情報の提供

本臨床研究の安全性に関する新たな情報を得た場合には、速やかに必要に応じて説明文書・同意文書の改定を行う。

10. 医療費の負担と補償

1）医療費

・初回の通常健診に掛かる費用は自己負担とし、当該試験によることが明らかな場合は患者自己負担はなしとする。不明瞭な場合は、協議をもって決定する。

・医療費が生じた際は、研究実施施設であるせんだい総合健診クリニックと本研究実施依頼会社である株式会社アドバンスト・メディカル・ケアにて協議し対応することとする。

2）補償

本臨床研究に起因して、被験者への健康被害の補償あるいは賠償責任が生じた場合、試験実施責任

医師および本研究実施依頼会社は協議の上、その取り扱いを決定する。

11. 倫理的事項

1）臨床研究に関する倫理指針の遵守

本臨床研究は、「臨床研究に関する倫理指針」（厚生労働省）に基づく倫理的原則、及び本臨床研究実施計画書を遵守して実施する。

2）倫理委員会

本試験は、「医療法人社団進興会 倫理審査委員会」において、当該試験計画の倫理性及び妥当性について審議する。試験は、委員会の承認を得た試験計画書に基づいて実施されなければならない。

① 重篤な有害事象が生じた場合

② 臨床研究実施計画書について重大な変更が行われる場合

③ 同意文書及びその他の説明文章について重大な変更が行われる場合

④ その他審査の対象となる資料が改定された場合

3）被験者の同意取得の方法

本臨床研究の開始に先立ち、臨床研究責任医師は被験者に対して下記の事項につき、説明文書に基づいて説明した後、被験者になることについての本人の自由意思に基づいた同意を文書により得る。

説明文書には以下の事項が記載されていること。

①研究を伴うこと

②臨床研究の目的

③臨床研究の方法（試験的側面、被験者の選択基準等を含む）

④被験者の臨床研究への参加予定期間

⑤臨床研究に参加する予定の被験者数

⑥予期される臨床上の利益及び危険性又は不便（被験者にとって予期される臨床上の利益がない場合はその旨を知らせること）

⑦患者を被験者にする場合における他の治療方法の有無及びその治療方法に関して予測される重要な利益及び危険性

⑧臨床研究に関連する健康被害が発生した場合に、被験者が受けることのできる治療

⑨臨床研究への参加は被験者の自由意思によるものであり、被験者は、被験者の臨床研究への参加を随時拒否又は撤回することができること。また、拒否あるいは撤回によって被験者が不利な扱いを受けたり、臨床研究に参加しない場合に受けるべき利益を失うことはないこと

⑩臨床研究への参加の継続について、被験者又はその代諾者の意思に影響を与える可能性のある情報が得られた場合には速やかに被験者に伝えること

⑪臨床研究への参加を中止させる場合の条件又は理由

⑫モニター、監査担当者、臨床研究審査委員会及び国内外の規制当局が診療録等の原資料を閲覧できること。その際、被験者の個人情報が保護されること。又、同意文書に被験者が記名捺印又は署名することによって閲覧を認めたことになること

⑬臨床研究の結果を公表する場合でも、被験者の個人情報は保護されること

⑭被験者が費用負担をする必要がある場合にはその内容

⑮臨床研究責任医師又は臨床研究協力者（コーディネーター）がいる場合はその氏名、職名及び連絡先

⑯被験者が臨床研究及び被験者の権利に関してさらに情報が欲しい場合に連絡をとるべき医療機関の相談窓口

⑰被験者が守るべき事項

4）被験者への情報の提供

臨床試験責任医師は、臨床研究への参加の継続について、被験者の意思に影響を与える可能性のある情報が得られた場合には、速やかに被験者又はその代諾者に伝え、臨床研究の参加を継続するか確認する。またこの経過を診療録に記載する。

5）個人情報の保護

臨床試験を実施するにあたっては、被験者の個人情報を保護する。

個人情報保護にあたっては、本試験に関わるデータを全て匿名化（コード化）し、取り扱うことを基本とする。尚、特定の患者個人が識別可能な情報については、せんだい総合健診クリニック個人情報管理責任者の厳密な管理のもと施錠できる場所にて保管管理する。不要になった際には焼却・溶解等、再現不能な形で処分する。尚、万一個人情報漏えいの可能性がある場合には速やかに以下の対応を行う。

－事実調査、原因の究明

－影響範囲の特定

－再発防止策の検討・実施

－影響を受ける可能性のある本人への連絡

－厚生労働大臣への報告

－事実関係、再発防止策等の公表

12. 試験実施施設

医療法人社団進興会 せんだい総合健診クリニック

13. 試験実施責任医師

石垣洋子（医療法人社団進興会せんだい総合健診クリニック院長）

吉形玲美（医療法人社団ミッドタウンクリニック 浜松町ハマサイトクリニック）

14. 個人情報管理責任者

川向由紀（医療法人社団進興会仙台業務課係長）

15. 臨床試験内容説明責任者

菊地恵観子（せんだい総合健診クリニック 仙台企画課 管理栄養士）

佐藤吏都子（せんだい総合健診クリニック 産業保険課 保健師）

16. 臨床試験事務局；

加藤　恵（せんだい総合健診クリニック 仙台企画課課長）

17. 検査キット

エクオールチェック：（株式会社ヘルスケアシステムズ）

腸内細菌叢検査：Mykinso（株式会社サイキンソー）

AGEｓ：AGE READER MU（セリスタ株式会社）

18. 披験食品の提供

(株)アドバンスト・メディカル・ケアより「エクオール+ラクトビオン酸」を提供

19. 研究成果の発表

本研究で得られた研究成果の発表に際しては、被験者の個人情報を保護する。

20. 費用概算

調査費用概算				
エクオールチェック	60例	2,700円/件	1回	¥162,000
腸内フローラ検査	60例	12,000円/件	1回	¥720,000
ファットスキャン	60例	5,000円/件	2回	¥600,000
baPWV	60例	5,000円/件	2回	¥600,000
エクオール提供	60例	-	（非摂取者にも試験終了後3本提供）	
AGE検査器具レンタル	60例	20,000円/日	2週間	¥280,000
食事アンケート	60例	50,000円		¥50,000
3ヶ月後血液検査	60例	（概算）10,000円/件	1回	¥600,000
合計				**¥3,012,000**

21. 参考文献

・Yoshikata R, et al, Relationship between equal producer status and metabolic parameters in 743 Japanese women: equal producer status is associated with antiatherosclerotic conditions in women around menopause and early postmenopause. Menopause 2017; 24, No2,216-224.

・Ishikawa N, et al, New equal supplement for relieving menopausal symptoms; randomized, placebo-controlled trial of Japanese women. Menopause 2009; 16:４1-148.

・Usui T, et al, Effects of natural E-equal supplements on overweight or obesity and metabolic syndrome in the Japanese, based on sex and equal status. Clin Endcrinol 2013; 78:365-372.

・Yoshizumi T, et al. Abdominal fat: standardized technique for measurement at CT. Radiology 1999; 211; 283-286.
