# Supplementary figures and images for: Effects of an equol-containing supplement on advanced glycation end products, visceral fat and climacteric symptoms in postmenopausal women: A randomized controlled trial
Source: PLoS One. 2021 Sep 10;16(9):e0257332. doi: 10.1371/journal.pone.0257332 (PMC8432832; doi:10.1371/journal.pone.0257332)

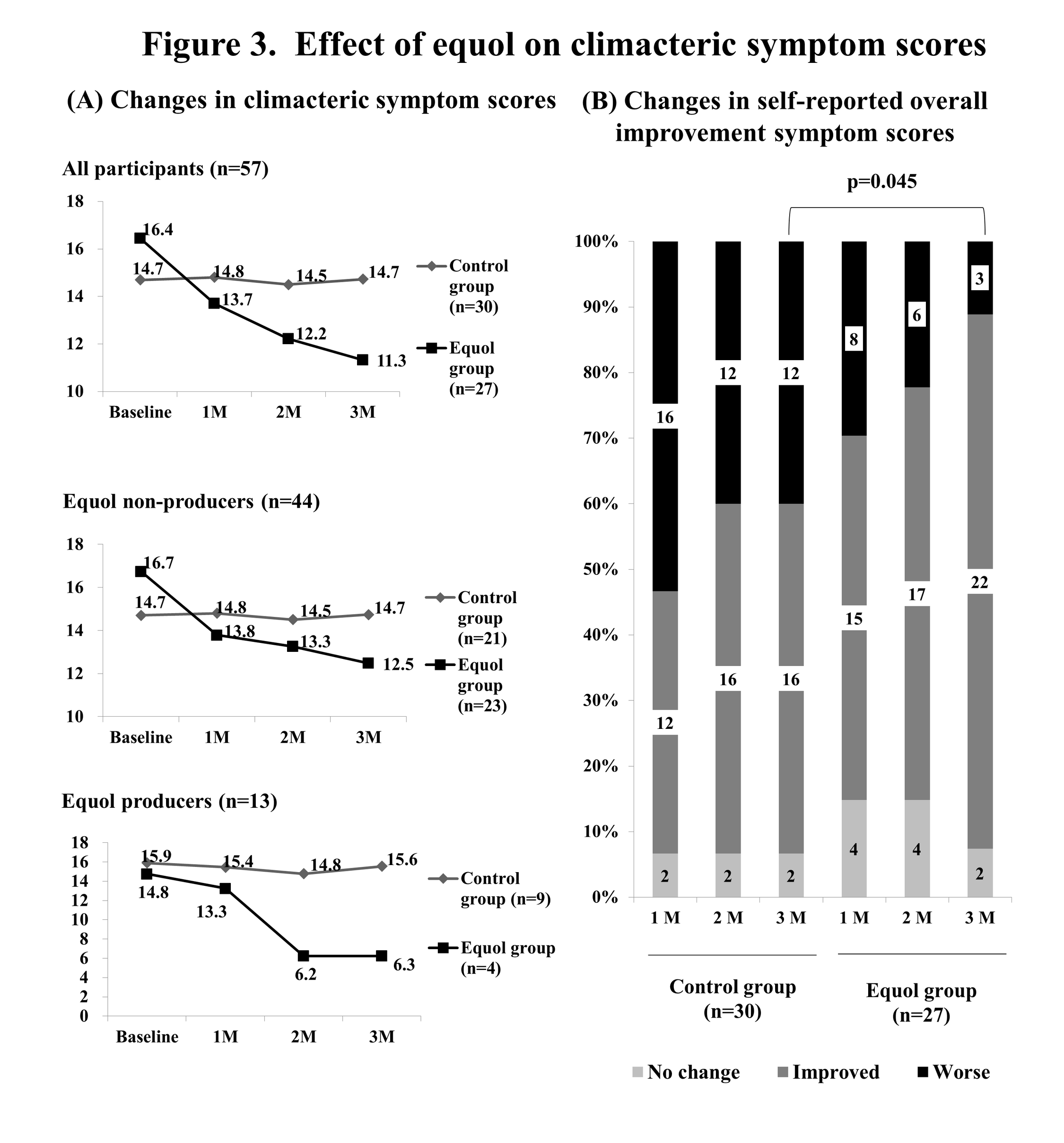

Supplement: S1 Data — (ZIP) [file pone.0257332.s004.zip › Fig3.tif]

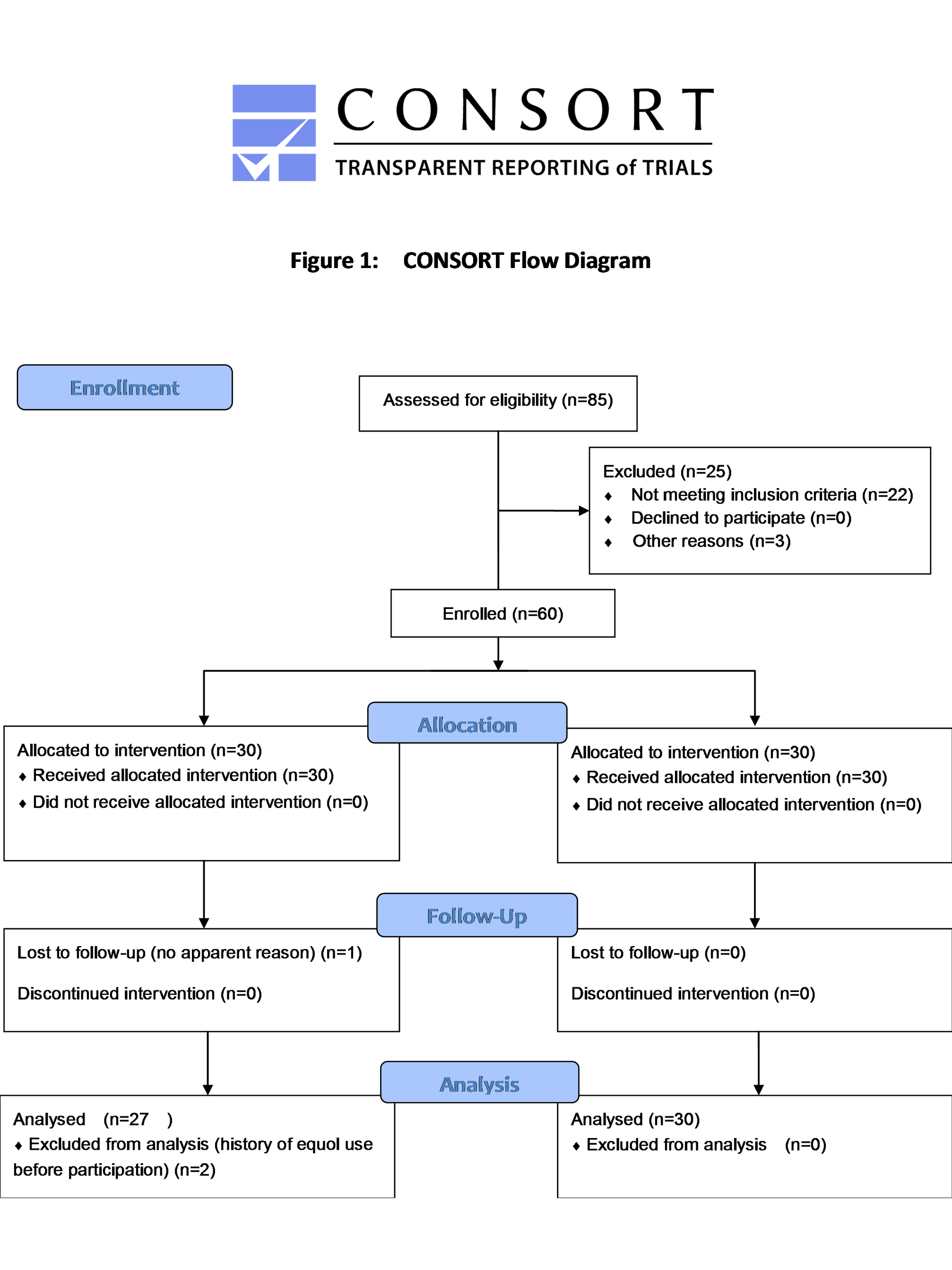

Supplement: S1 Data — (ZIP) [file pone.0257332.s004.zip › Fig1.tif]

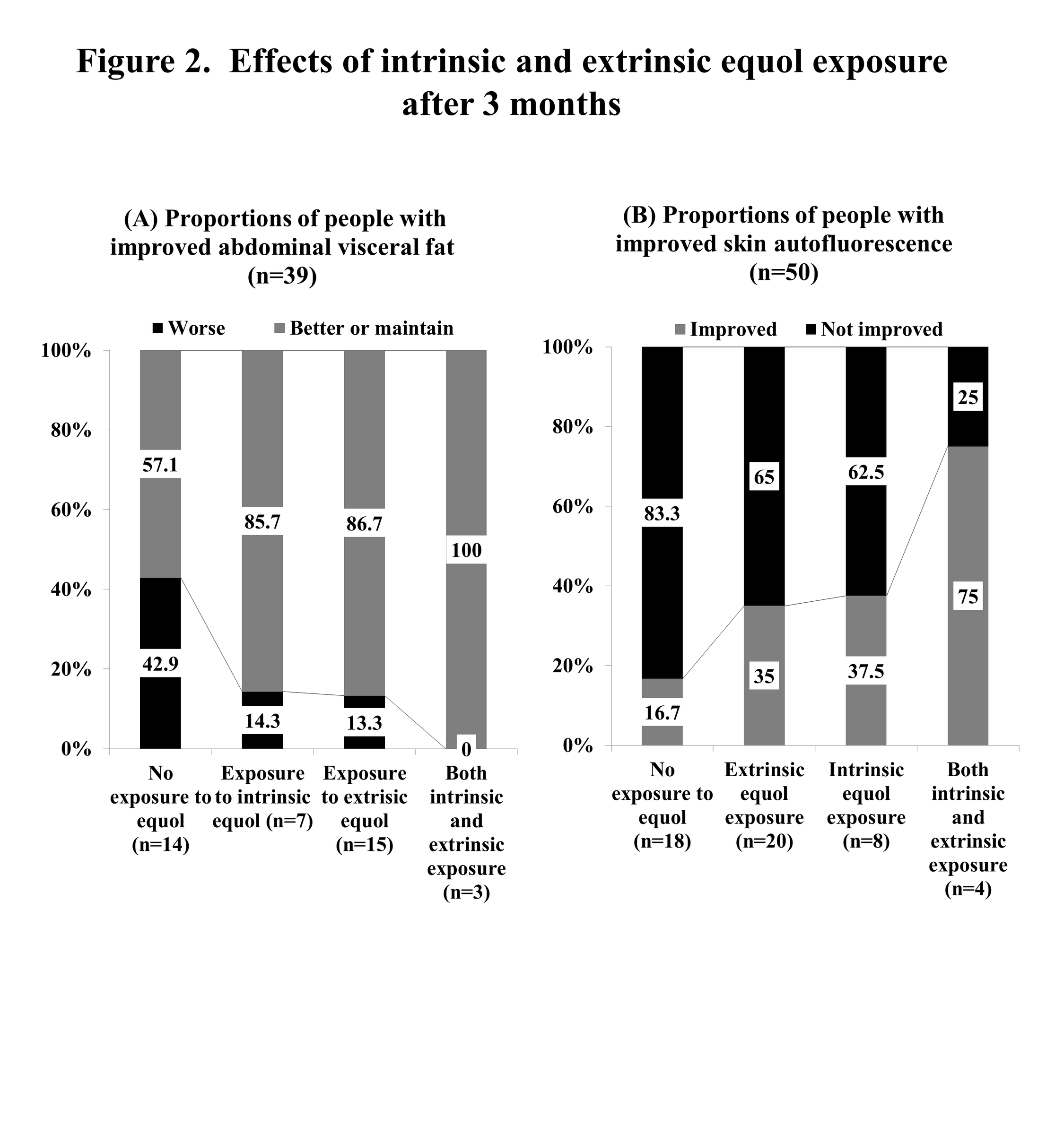

Supplement: S1 Data — (ZIP) [file pone.0257332.s004.zip › Fig2.tif]
