# Supplementary material for: Effects of an equol-containing supplement on advanced glycation end products, visceral fat and climacteric symptoms in postmenopausal women: A randomized controlled trial
Source: PLoS One. 2021 Sep 10;16(9):e0257332. doi: 10.1371/journal.pone.0257332 (PMC8432832; doi:10.1371/journal.pone.0257332)
Supplement: Attachment — Submitted filename: Response to Reviewers.docx [file pone.0257332.s005.docx]

1. Please ensure that your manuscript meets PLOS ONE's style requirements, including those for file naming. The PLOS ONE style templates can be found at

<https://journals.plos.org/plosone/s/file?id=wjVg/PLOSOne_formatting_sample_main_body.pdf> and

https://journals.plos.org/plosone/s/file?id=ba62/PLOSOne_formatting_sample_title_authors_affiliations.pdf

 🡪We have revised accordingly.

2.We note that you have indicated that data from this study are available upon request. PLOS only allows data to be available upon request if there are legal or ethical restrictions on sharing data publicly. For information on unacceptable data access restrictions, please see <http://journals.plos.org/plosone/s/data-availability#loc-unacceptable-data-access-restrictions>.

 In your revised cover letter, please address the following prompts:

a) If there are ethical or legal restrictions on sharing a de-identified data set, please explain them in detail (e.g., data contain potentially identifying or sensitive patient information) and who has imposed them (e.g., an ethics committee). Please also provide contact information for a data access committee, ethics committee, or other institutional body to which data requests may be sent.

b) If there are no restrictions, please upload the minimal anonymized data set necessary to replicate your study findings as either Supporting Information files or to a stable, public repository and provide us with the relevant URLs, DOIs, or accession numbers. Please see <http://www.bmj.com/content/340/bmj.c181.long> for guidelines on how to de-identify and prepare clinical data for publication. For a list of acceptable repositories, please see <http://journals.plos.org/plosone/s/data-availability#loc-recommended-repositories>.

We will update your Data Availability statement on your behalf to reflect the information you provide.

   🡪We have uploaded the minimal anonymized data set in Harvard Dataverse and provided the link.

3. Please amend either the title on the online submission form (via Edit Submission) or the title in the manuscript so that they are identical.

   🡪We have revised accordingly.

4. Your ethics statement should only appear in the Methods section of your manuscript. If your ethics statement is written in any section besides the Methods, please move it to the Methods section and delete it from any other section. Please ensure that your ethics statement is included in your manuscript, as the ethics statement entered into the online submission form will not be published alongside your manuscript.

🡪We have moved it from the declaration section to the method section.

5. Please include captions for your Supporting Information files at the end of your manuscript, and update any in-text citations to match accordingly. Please see our Supporting Information guidelines for more information: http://journals.plos.org/plosone/s/supporting-information.

 🡪We have moved it from the declaration section to the method section (line 88-93).

6.Thank you for stating the following financial disclosure:

 "Advanced Medical Care Co. Ltd."

Please state what role the funders took in the study.  If the funders had no role, please state: "The funders had no role in study design, data collection and analysis, decision to publish, or preparation of the manuscript."

If this statement is not correct you must amend it as needed.

Please include this amended Role of Funder statement in your cover letter; we will change the online submission form on your behalf.

🡪We have included this amended Role of Funder statement in our cover letter.

7. Thank you for stating the following in the Competing Interests section:

"Advanced Medical Care Co. Ltd. provides administrative support to Tokyo Midtown Medical Center."

Please confirm that this does not alter your adherence to all PLOS ONE policies on sharing data and materials, by including the following statement: "This does not alter our adherence to  PLOS ONE policies on sharing data and materials.” (as detailed online in our guide for authors http://journals.plos.org/plosone/s/competing-interests).  If there are restrictions on sharing of data and/or materials, please state these. Please note that we cannot proceed with consideration of your article until this information has been declared.

 Please include your updated Competing Interests statement in your cover letter; we will change the online submission form on your behalf.

🡪We have included this Competing Interests statement in our cover letter.

Please know it is PLOS ONE policy for corresponding authors to declare, on behalf of all authors, all potential competing interests for the purposes of transparency. PLOS defines a competing interest as anything that interferes with, or could reasonably be perceived as interfering with, the full and objective presentation, peer review, editorial decision-making, or publication of research or non-research articles submitted to one of the journals. Competing interests can be financial or non-financial, professional, or personal. Competing interests can arise in relationship to an organization or another person. Please follow this link to our website for more details on competing interests: http://journals.plos.org/plosone/s/competing-interests

[Note: HTML markup is below. Please do not edit.]

**Comments to the Author**
5. Review Comments to the Author
Please use the space provided to explain your answers to the questions above. You may also include additional comments for the author, including concerns about dual publication, research ethics, or publication ethics. (Please upload your review as an attachment if it exceeds 20,000 characters)

 Reviewer #1: A two-arm randomized controlled study (n=57) was conducted to compare pre and post intervention of Equol on climacteric symptoms in post-menopausal women. At 3-month follow-up the intervention group showed statistically significant improvements in climacteric symptoms compared to placebo.
Minor revisions:
1- Table 2: Typographical error in footnote: Replace A with a

🡪We have revised accordingly.
2- Line 212: Provide 95% confidence intervals for the proportions 26.7% and 3.7%.

🡪 We have provided the 95% confidence interval for the proportions 26.7% and 3.7% in line 221 as follows.

Post 3 months, more women in the control group were found to have worsen PWV values compared to those in the equol intervention group (26.7% vs. 3.7%, x-squared (2, 27) = 6.33, 95% CI [0.056, 0.403], p=0.042).
3- Table 3: Typographical error in footnote: Mann-Whitney test.

🡪We have revised accordingly.
4- Indicate if any adverse events occurred during the course of the study.

🡪We added information on the adverse events during the course of the study in line 172 as follows.

No participant reported any adverse effects during the course of the study.
5- State and justify the study’s target sample size with a pre-study statistical power calculation. The power calculation should include: sample size, alpha level (indicating one or two-sided), minimal detectable difference and statistical testing method.

🡪We have state and justified the study’s target sample size with a pre-study statistical power calculation in line 371-373 as follows.

A post hoc power analysis was conducted using R statistical software 4.1. The analysis revealed that the statistical power of t- test was 95% for a moderate effect size of 0.5 for the sample size of 27 in each group, as significant at the 5% level (two tailed).

Reviewer #2: Paper titled (Equol effects on AGE skin products, visceral fat, and climacteric symptoms in postmenopausal women) by Yoshikata et al. demonstrated the effect of Equol, an isoflavone derivative with similar chemical structure like estrogen.
Although this is an interesting topic with positive outcome. However, the style of writing is hard to be comprehended or followed.
This was starting from the Title until the end of Ms
For example: title: Equol effect on.... : this is better saif The effect of Equol, an isoflavone, on .....
This is since this flavone is not very common or known.
Kindly consult a colleague proficient in English and academic writing to help you better demonstrate your idea

🡪We have submitted the revised manuscript to the academic editing service for language edit.
